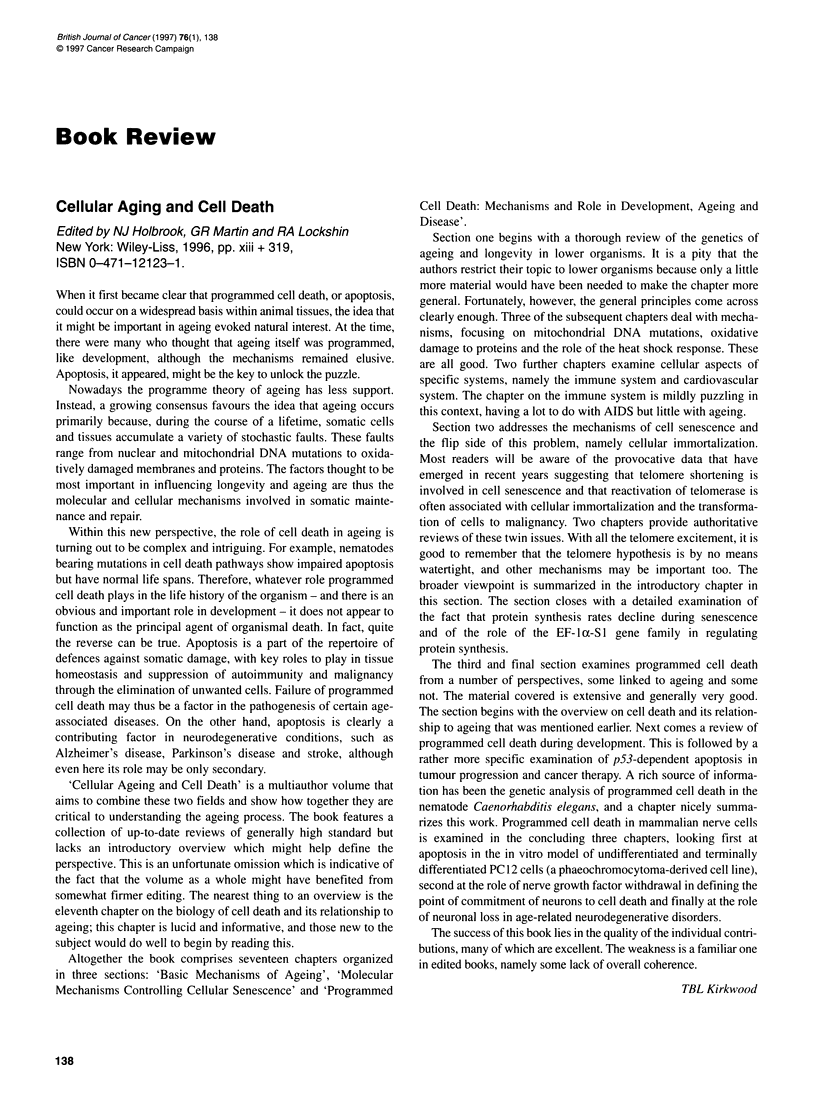# Cellular Aging and Cell Death

**Published:** 1997

**Authors:** TBL Kirkwood


British Journal of Cancer (1997) 76(1), 138
© 1997 Cancer Research Campaign

### Book Review

### Cellular Aging and Cell Death

*Edited by NJ Holbrook, GR Martin and RA Lockshin*
New York: Wiley-Liss, 1996, pp. xiii + 319,
ISBN 0–471–12123–1.

When it first became clear that programmed cell death, or apoptosis, could occur on a widespread basis within animal tissues, the idea that it might be important in ageing evoked natural interest. At the time, there were many who thought that ageing itself was programmed, like development, although the mechanisms remained elusive. Apoptosis, it appeared, might be the key to unlock the puzzle.

Nowadays the programme theory of ageing has less support. Instead, a growing consensus favours the idea that ageing occurs primarily because, during the course of a lifetime, somatic cells and tissues accumulate a variety of stochastic faults. These faults range from nuclear and mitochondrial DNA mutations to oxidatively damaged membranes and proteins. The factors thought to be most important in influencing longevity and ageing are thus the molecular and cellular mechanisms involved in somatic maintenance and repair.

Within this new perspective, the role of cell death in ageing is turning out to be complex and intriguing. For example, nematodes bearing mutations in cell death pathways show impaired apoptosis but have normal life spans. Therefore, whatever role programmed cell death plays in the life history of the organism – and there is an obvious and important role in development – it does not appear to function as the principal agent of organismal death. In fact, quite the reverse can be true. Apoptosis is a part of the repertoire of defences against somatic damage, with key roles to play in tissue homeostasis and suppression of autoimmunity and malignancy through the elimination of unwanted cells. Failure of programmed cell death may thus be a factor in the pathogenesis of certain age-associated diseases. On the other hand, apoptosis is clearly a contributing factor in neurodegenerative conditions, such as Alzheimer's disease, Parkinson's disease and stroke, although even here its role may be only secondary.

'Cellular Ageing and Cell Death' is a multiauthor volume that aims to combine these two fields and show how together they are critical to understanding the ageing process. The book features a collection of up-to-date reviews of generally high standard but lacks an introductory overview which might help define the perspective. This is an unfortunate omission which is indicative of the fact that the volume as a whole might have benefited from somewhat firmer editing. The nearest thing to an overview is the eleventh chapter on the biology of cell death and its relationship to ageing; this chapter is lucid and informative, and those new to the subject would do well to begin by reading this.

Altogether the book comprises seventeen chapters organized in three sections: 'Basic Mechanisms of Ageing', 'Molecular Mechanisms Controlling Cellular Senescence' and 'Programmed Cell Death: Mechanisms and Role in Development, Ageing and Disease'.

Section one begins with a thorough review of the genetics of ageing and longevity in lower organisms. It is a pity that the authors restrict their topic to lower organisms because only a little more material would have been needed to make the chapter more general. Fortunately, however, the general principles come across clearly enough. Three of the subsequent chapters deal with mechanisms, focusing on mitochondrial DNA mutations, oxidative damage to proteins and the role of the heat shock response. These are all good. Two further chapters examine cellular aspects of specific systems, namely the immune system and cardiovascular system. The chapter on the immune system is mildly puzzling in this context, having a lot to do with AIDS but little with ageing.

Section two addresses the mechanisms of cell senescence and the flip side of this problem, namely cellular immortalization. Most readers will be aware of the provocative data that have emerged in recent years suggesting that telomere shortening is involved in cell senescence and that reactivation of telomerase is often associated with cellular immortalization and the transformation of cells to malignancy. Two chapters provide authoritative reviews of these twin issues. With all the telomere excitement, it is good to remember that the telomere hypothesis is by no means watertight, and other mechanisms may be important too. The broader viewpoint is summarized in the introductory chapter in this section. The section closes with a detailed examination of the fact that protein synthesis rates decline during senescence and of the role of the EF-1α-S1 gene family in regulating protein synthesis.

The third and final section examines programmed cell death from a number of perspectives, some linked to ageing and some not. The material covered is extensive and generally very good. The section begins with the overview on cell death and its relationship to ageing that was mentioned earlier. Next comes a review of programmed cell death during development. This is followed by a rather more specific examination of *p53*-dependent apoptosis in tumour progression and cancer therapy. A rich source of information has been the genetic analysis of programmed cell death in the nematode *Caenorhabditis elegans*, and a chapter nicely summarizes this work. Programmed cell death in mammalian nerve cells is examined in the concluding three chapters, looking first at apoptosis in the in vitro model of undifferentiated and terminally differentiated PC12 cells (a phaeochromocytoma-derived cell line), second at the role of nerve growth factor withdrawal in defining the point of commitment of neurons to cell death and finally at the role of neuronal loss in age-related neurodegenerative disorders.

The success of this book lies in the quality of the individual contributions, many of which are excellent. The weakness is a familiar one in edited books, namely some lack of overall coherence.

*TBL Kirkwood*